# Effects of *Monascus pilosus* SWM 008-Fermented Red Mold Rice and Its Functional Components on Gut Microbiota and Metabolic Health in Rats

**DOI:** 10.3390/foods14040651

**Published:** 2025-02-14

**Authors:** Pei-Xin Yang, Chen-Ru You, Yun-Hsuan Lin, Chia-Shu Wang, Ya-Wen Hsu, Tzu-Ming Pan, Chun-Lin Lee

**Affiliations:** 1 Department of Life Science, National Taitung University, Taitung 95092, Taiwan; px.yang@sunway.cc (P.-X.Y.);; 2 SunWay Biotech Co., Taipei 11494, Taiwan; bill.wang@sunway.cc (C.-S.W.); hyw@sunway.cc (Y.-W.H.); 3 Department of Biochemical Science and Technology, National Taiwan University, Taipei 10617, Taiwan

**Keywords:** *Monascus pilosus*, gut microbiota, monascin, monascinol, ankaflavin, polysaccharides

## Abstract

Red mold rice, fermented by *Monascus* spp., has been reported to modulate gut microbiota composition and improve metabolic health. Previous studies indicate that red mold rice can reduce cholesterol, inhibit hepatic lipid accumulation, and enhance bile acid excretion, while also altering gut microbiota under high-fat dietary conditions. However, it remains unclear whether these effects are directly due to *Monascus*-derived products modulating gut microbiota or are a consequence of improved metabolic health conditions, which indirectly influence gut microbiota. This study aimed to evaluate the effects of *Monascus pilosus* SWM 008 fermented red mold rice and its components—monascin, monascinol, ankaflavin, and polysaccharides—on gut microbiota and metabolic health in rats fed a normal diet. Over eight weeks, physiological, biochemical, and gut microbiota parameters were assessed. Results showed no significant changes in body weight or liver/kidney function, confirming safety. Gut microbiota analysis revealed that red mold rice, monascin, monascinol, and polysaccharides significantly altered gut microbiota composition by increasing the relative abundance of beneficial bacteria, such as *Akkermansia muciniphila*, *Ligilactobacillus murinus*, and *Duncaniella dubosii*. Functional predictions indicated enhanced vitamin K2 biosynthesis, nucleotide metabolism, and other metabolic pathways linked to improved gut health. In conclusion, *Monascus pilosus* SWM 008 fermented red mold rice demonstrated safety and beneficial effects, suggesting its potential as a functional food to maintain gut microbiota balance under normal dietary conditions.

## 1. Introduction

Cardiovascular disease and metabolic syndrome are significant global health concerns, often exacerbated by lifestyle factors such as poor diet and physical inactivity. These conditions are characterized by a combination of obesity, hypertension, dyslipidemia, and glucose intolerance, leading to an increased risk of cardiovascular events and type 2 diabetes. Functional foods and natural compounds have emerged as promising interventions to modulate these risk factors and improve overall metabolic health. Among these, compounds derived from *Monascus* species, such as monascin, ankaflavin, and monascinol, have gained increasing attention for their potential cardiometabolic benefits.

*Monascus*-fermented red mold rice along with its bioactive compounds like monascin, ankaflavin, and monascinol, has been extensively studied for its lipid-lowering and cardiovascular protective effects. *Monascus pilosus* SWM 008 is a strain known for producing monascinol and monascin. Previous research has highlighted the roles of monascin and ankaflavin in reducing body fat, improving blood pressure, and enhancing lipid profiles. Clinical studies, such as those by Chen [1] and Liu [2], demonstrated that Ankascin 568, a *Monascus purpureus* NTU 568-fermented product, significantly reduced total cholesterol (TC), low-density lipoprotein cholesterol (LDL-C), and blood pressure while improving high-density lipoprotein cholesterol (HDL-C) levels. These findings indicate substantial cardiometabolic benefits and suggest a potential role for these compounds in managing cardiovascular disease risk factors.

In addition to monascin and ankaflavin, recent studies have shown that monascinol exhibits potent hypolipidemic effects, surpassing monascin in reducing fatty acid synthase (FASN) and sterol regulatory element-binding protein 2 (SREBP2) expression while promoting triglyceride and bile acid excretion [3]. This suggests that monascinol may offer additional benefits in regulating lipid metabolism and reducing liver inflammation, further highlighting the potential of *Monascus*-derived compounds as functional ingredients for metabolic health.

Gut microbiota plays a crucial role in host metabolism, immunity, and overall health. Specific microbial taxa, such as Firmicutes, Bacteroidota, *Akkermansia muciniphila*, and *Ligilactobacillus murinus*, have been associated with metabolic health. An increased Firmicutes/Bacteroidota ratio has been linked to obesity and metabolic disorders, while Bacteroidota fermentation produces short-chain fatty acids (SCFAs) that contribute to anti-inflammatory effects and improved energy metabolism. *Akkermansia muciniphila*, a key probiotic, has been shown to strengthen the gut barrier and reduce inflammation, playing a protective role in conditions like obesity and type 2 diabetes [4]. Thus, modulating gut microbiota through functional foods such as red mold rice could offer a novel approach to improving metabolic health.

Moreover, red mold rice, fermented by *Monascus*, has been reported to alter gut microbiota composition, contributing to improved metabolic outcomes. Studies have demonstrated that red mold rice can reduce cholesterol and LDL-C, inhibit hepatic lipid accumulation, and enhance cholesterol and bile acid excretion, while also modulating gut microbiota to counteract high-fat diet-induced dysbiosis [5,6]. Although these studies suggest that *Monascus*-fermented products and their components may regulate gut microbiota under high-fat dietary conditions, it remains uncertain whether the observed modulation of gut microbiota is a direct result of the *Monascus*-fermented products themselves or an indirect consequence of improved disease conditions.

This study aims to evaluate the effects of *Monascus*-fermented red mold rice, specifically fermented by *Monascus pilosus* SWM 008, and its key bioactive components, including monascin, monascinol, and polysaccharide on gut microbiota composition under non-high-fat dietary conditions. The study also explores the effects of ankaflavin, a key metabolite of *Monascus purpureus* NTU 568, to better understand their individual and combined impacts on gut health. This experiment was modified based on the study by Zhou et al. [6], using high-throughput sequencing to analyze changes in gut microbiota, including alterations in microbial abundance, species difference statistical analysis, and functional prediction and correlation analysis. This study seeks to elucidate the prebiotic potential and microbiota-modulating effects of red mold rice and its functional components in a standard dietary context. Specifically, it seeks to determine whether red mold rice and its components directly influence gut microbiota composition or whether the changes in gut microbiota are secondary to improvements in metabolic health. The findings will provide insights into the broader health implications of red mold rice beyond its application in high-fat diet models, potentially supporting its use as a functional food for metabolic health and microbiota modulation.

## 2. Materials and Methods

### 2.1. Chemical and Substance

This study substances were obtained by SunWay Biotech. Co. Ltd. (Taipei, Taiwan), utilizing the *Monascus pilosus* SWM 008 and *Monascus purpureus* NTU 568 strain to perform large-scale fermentation of 200 kg of red mold rice. Msol and MS were extracted and purified from *M. pilosus* SWM 008, while AK was extracted and purified from *M. purpureus* NTU 568. The extraction and purification methods were modified as per Hsu et al. [7]. Specifically, dried red mold rice (5 kg) was soaked and extracted twice with 25 L of 95% edible ethanol at 60 °C for 2 h. The extracts were collected and concentrated under reduced pressure to remove ethanol. The dried residue was separated twice using silica gel column chromatography to obtain partially purified fractions. These fractions were further purified using semi-preparative HPLC to obtain the standard compounds. The purity of the materials was confirmed via high-performance liquid chromatography (HPLC) (Shimadzu, Kyoto, Japan), with final purities of MS, Msol, and AK determined to be 99%, 98%, and 97%, respectively (chromatograms are provided in the Supplementary Data). The fermented rice used contains no citrinin, with 1 g of SWM 008 fermented rice providing 3 mg of monascinol, 6 mg of monascin, and 17.7 mg of polysaccharides. Based on previous experimental results and literature recommendations, the suggested daily intake of SWM 008 fermented rice is 1 g [3].

### 2.2. Animal Housing and Experimental Model

This study utilized 6-week-old male Sprague Dawley rats obtained from Bio-Lasco Biotechnology Co. (Taipei, Taiwan), with eight rats per group. The animals were housed in a controlled environment at 23 ± 1 °C with a 12 h light/dark cycle from 8:00 to 20:00. Food and water were provided ad libitum. After one week of acclimatization, rats were administered a fixed dose of the test substance via oral gavage for eight weeks. The dosage was calculated according to the US FDA guidelines, based on the human equivalent dose for a 60 kg adult, at 6.2 times the recommended human intake per kg body weight [8]. Body weight, food intake, and water intake were monitored weekly throughout the experiment. During the entire study period, the animals were allowed unrestricted access to food. At the end of the experiment, caloric intake and feed efficiency ([weight gain/food intake] × 100%) were estimated based on food consumption.

The animal dosages for each group were calculated based on the recommended human dosages to ensure appropriate scaling. The experimental groups are as follows: NOR: This group serves as the negative control, receiving only reverse osmosis (RO) water without any active experimental substance. R1X: This group is administered red mold rice at a dosage equivalent to 1 g per 60 kg of body weight per day in humans, which corresponds to a rat dose of 0.103 g/kg body weight per day. R5X: In this high-dose group, red mold rice is administered at a dosage equivalent to 5 g per 60 kg of body weight per day in humans, corresponding to 0.517 g/kg body weight per day in rats. PS: This group is administered *Monascus* polysaccharide at a dosage equivalent to 0.2 g per 60 kg of body weight per day in humans, corresponding to 0.021 g/kg body weight per day in rats. MS: monascin is administered at a dosage equivalent to 0.003 g per 60 kg of body weight per day in humans, corresponding to 0.00031 g/kg body weight per day in rats. AK: This group is administered ankaflavin at a dosage equivalent to 0.003 g per 60 kg of body weight per day in humans, which corresponds to 0.00031 g/kg body weight per day in rats. Msol: monascinol is administered at a dosage equivalent to 0.003 g per 60 kg of body weight per day in humans, corresponding to 0.00031 g/kg body weight per day in rats. After 8 weeks, experimental animals were fasted for 16 h before being euthanized via carbon dioxide asphyxiation. Once cessation of heartbeat and respiration was confirmed, dissection commenced, and blood, liver, and kidney tissues, as well as ileum segments, were collected. The liver and kidney were weighed, and sections of the liver’s second largest lobe, the left kidney and ileum, were preserved in 10% formalin for pathological use. The remaining liver tissue was washed with 0.9% saline, and the residual right kidney and ileum segments were stored in zip-lock bags at −80 °C. Blood samples were allowed to settle for 2 h, after which they were centrifuged at 6000× *g* for 15 min to separate the serum, which was then stored at −80 °C.

### 2.3. Serum Biochemical Analysis

At the end of the experimental period, serum samples were collected from the test animals for biochemical analysis. The analysis aimed to assess various aspects including carbohydrate metabolism, and liver and kidney function. Specifically, the parameters measured included alanine aminotransferase (ALT), aspartate aminotransferase (AST), creatine phosphokinase (CPK), TC, triglycerides (TG), uric acid, blood urea nitrogen (BUN), coagulation factor II. They were determined using a biochemical autoanalyzer (c702, Roche, Basel, Switzerland).

### 2.4. Hematoxylin and Eosin Stain

Liver, kidney, and ileum tissue samples were fixed in 10% formaldehyde, dehydrated, and embedded in paraffin, with sections stained using hematoxylin and eosin (H&E) for histological analysis. The slides were examined under a microscope at 400× magnification to evaluate tissue morphology.

### 2.5. 16S Gut Microbiota Analysis

The study also sought to evaluate the safety and effects of red mold rice, monascinol, and monascin on gut microbiota. Fecal samples were collected from the test animals at the 8th week to perform 16S microbiota analysis, which included assessment of species distribution (relative abundance), sample complexity (alpha diversity), comparative analysis among groups (beta diversity), and statistical analysis of group differences, thereby investigating the ability of these substances to modulate the intestinal microecological balance.

#### 2.5.1. Genomic DNA Extraction and PCR Amplification

Total genomic DNA was extracted from the samples using the QIAamp PowerFecal DNA kit (Qiagen Co., Hilden, Germany). The DNA concentration was determined with a Qubit 4.0 fluorometer (Thermo Fisher Scientific Co., Waltham, MA, USA) and adjusted to 1 ng/µL. The full-length 16S gene (V1-V9 region) was amplified using barcode-labeled 16S-specific primers (Forward: 5′Phos/GCATC-16-base barcode-AGRGTTYGATYMTGGCTCAG-3′, Reverse: 5′Phos/GCATC-16-base barcode-RGYTACCTTGTTACGACTT-3′). PCR was performed using KAPA HiFi HotStart ReadyMix (Roche Co., Basel, Switzerland) under the following conditions: initial denaturation at 95 °C for 3 min; 20–27 cycles (depending on sample) of 95 °C for 30 s, 57 °C for 30 s, and 72 °C for 60 s; with a final extension at 72 °C for 5 min. The PCR products were monitored on 1% agarose gel, and samples with a bright main band around 1500 bp were selected using AMPure PB Beads and purified for SMRTbell library preparation.

#### 2.5.2. SMRTbell Library Construction and Sequencing

The SMRTbell library was constructed for full-length 16S gene amplification using barcode primers for multiplex SMRTbell library preparation and sequencing (PacBio Co., Menlo park, CA, USA). Sequencing was performed on a PacBio Sequel IIe system using the circular consensus sequencing (CCS) mode, generating HiFi reads with predicted accuracy (Phred Scale) ≥ 30. The target DNA underwent repeated sequencing, and errors were corrected by comparing the results of repeated sequencing to achieve a final high accuracy of >99.9% (QV30).

#### 2.5.3. Bioinformatics Analysis

Sequence Data Processing. The sequencing data were demultiplexed based on the barcode sequences and PCR primer sequences and then trimmed to remove the barcodes and primers. The resulting sequences were imported into QIIME2 (v2019.7.0; https://qiime2.org/,1 June2023) [9] to create an artifact containing all the sequences. QIIME2 cutadapt was used to remove the amplicon variant primers (forward primer: CCTACGGGNGGCWG; reverse primer: GACTACHVGGGTATCTAATCC), and the trimmed sequences were used as input reads for QIIME2 DADA2.

QIIME2 DADA2 Microbiota Analysis. QIIME2 DADA2 (v2019.7.0; https://qiime2.org/) [10,11] was used for denoising, starting with filtering sequences and trimming them to a specified length (forward reads: 280 bp; reverse reads: 220 bp) while setting the MaxEE (forward reads: 2; reverse reads: 2) parameter to limit the maximum expected number of errors per sequence. The DADA2 core algorithm was then applied to correct base errors using sequence abundance, quality scores, and relationships between sequences to infer the true sequences. After denoising each of the paired-end sequences, they were merged (minimum overlap length of 20 bp; no mismatches allowed in the overlap region). Chimeras were removed by comparing sequences of lower abundance to those with higher abundance. The resulting sequences were identified as Amplicon Sequence Variants (ASVs), which were used to generate the ASV table for downstream species annotation. QIIME2 feature-classifier (v2019.7.0; https://qiime2.org/) [12] and the classify-sklearn machine learning method [13,14] trained with various databases and specific variant regions were used for species-level annotation of ASVs, providing taxonomic information at the kingdom, phylum, class, order, family, genus, and species levels. For 16S analysis, QIIME2 alignment MAFFT method (v2019.7.0; https://qiime2.org/) [15,16] and the GreenGenes database (gg_13_8) [17,18] or Silva database (v132; 2017.12) [19] were used for rapid sequence alignment. Additionally, the latest NCBI database was used to obtain annotation information [20,21,22]. For ITS analysis, the Unite database (v7.2; 2017.12.01) [23,24,25,26] was used for annotation. Sequence data from each sample were normalized by the smallest number of tags among all samples [27], and the normalized data were used for subsequent alpha and beta diversity analysis.

#### 2.5.4. Alpha Diversity

Analysis Alpha diversity indices, including Observed-species, Shannon, Simpson, ACE, Chao1, PD_whole_tree, and Goods_coverage, were calculated using Qiime, and rarefaction curves, rank abundance curves, species accumulation curves, and between-group alpha diversity analyses were plotted using R (v3.3.1). Rarefaction curves displayed the number of observed ASVs as a function of sequencing depth, providing insights into species richness. Rank abundance curves illustrated species richness and evenness by plotting ASVs ranked by abundance against their relative abundance, where curve width and flatness reflected richness and evenness, respectively. Boxplots were used to visualize the distribution of alpha diversity indices (Observed Species and Shannon Index) among treatment groups. Group differences were assessed using non-parametric Kruskal–Wallis and Wilcoxon tests.

#### 2.5.5. Beta Diversity Analysis

Beta diversity analysis was performed focusing on Constrained Principal Coordinates Analysis (CPCoA), Partial Least Squares Discriminant Analysis (PLS-DA), UniFrac distances, and UPGMA clustering trees. UniFrac distances were calculated in Qiime to assess phylogenetic differences between microbial communities, and UPGMA clustering trees were constructed to illustrate sample clustering patterns. CPCoA was conducted to visualize group differentiation, with the X and Y axes representing the first two components and their contribution to sample variation. PLS-DA was performed using the mixOmics package in R to evaluate group classification effectiveness, plotting each sample based on its predicted group membership.

#### 2.5.6. Species Difference Statistical Analysis

Species difference analysis was conducted using LEfSe and MetagenomeSeq to identify significant taxa differences between the experimental and control groups. LEfSe analysis was performed with an LDA score threshold of 2.0 to determine taxa with significant effects, and the results were visualized using bar plots and cladograms. The length of each bar represented the impact size, with colors indicating the group where taxa were enriched. MetagenomeSeq analysis focused on key genera, such as *Akkermansia*, and used the zero-inflated log-normal model (fitZig) to account for sequencing depth variations. Statistical significance was determined using an FDR-adjusted *p*-value threshold of 0.05, and box plots were used to highlight genus-level differences in microbial abundance. Species difference analysis was performed using Welch’s *t*-test in STAMP (v2.1.3) to generate bar plots showing intergroup species differences. MetagenomeSeq analysis was conducted using the metagenomeSeq package in R, applying a permutation test across different taxonomic levels (Phylum, Class, Order, Family, Genus, Species) to obtain *p*-values, which were then adjusted using the Benjamini and Hochberg false discovery rate method to obtain *q*-values. Box plots of relative abundance distributions were generated for taxa with significant intergroup differences (*p*/*q* < 0.05). LEfSe analysis was performed with the LDA score threshold set to 4 to identify significantly different taxa. The analysis included a bar plot displaying LDA scores, with the length of each bar indicating the impact size of the taxon, and a cladogram showing significant taxa across multiple taxonomic levels. ANOSIM, MRPP, and Adonis analyses were conducted using the anosim, mrpp, and adonis functions in the R vegan package, respectively. Intergroup differential species analysis was also performed using t-tests in R. Dominant species Spearman correlation analysis was conducted using the corrplot package in R.

#### 2.5.7. Functional Prediction and Correlation Analysis

Functional prediction of gut microbiota was carried out using PICRUSt2 to evaluate changes in metabolic pathways. Functional pathways significantly increased or decreased across treatment groups compared to the NOR group were identified, with significance assessed using Welch’s *t*-test and Bonferroni correction (*p* < 0.05). Results were visualized with red and blue squares to indicate significant and non-significant changes, respectively. Correlation and redundancy analyses were performed to explore relationships between the top 20 genera, predicted metabolic pathways, and experimental factors. Heatmaps were used to display the correlation between genera and pathways, while correlograms illustrated genus-function relationships with red and blue lines representing positive and negative correlations. Redundancy Analysis (RDA) was conducted to integrate the top 20 genera, predicted metabolic pathways, and experimental factors, with Pearson correlation and FDR-adjusted *p*-values applied to assess statistical significance. PICRUSt2 was used to predict the functional pathways of gut microbiota based on 16S rRNA gene sequences. Functional pathways significantly increased or decreased in treatment groups compared to the NOR group were identified. Welch’s T-test with Bonferroni correction was applied to determine statistical significance (*p* < 0.05). Correlation analysis and RDA were performed to explore relationships between the top 20 genera, predicted metabolic pathways, and experimental factors. Heatmaps were used to visualize correlations between genera and predicted pathways or experimental factors, and correlograms illustrated genus-function relationships with red and blue lines indicating positive and negative correlations, respectively. Constrained ordination analysis was conducted using RDA to integrate genera, pathways, and experimental factors. Statistical significance was assessed using Pearson correlation and FDR-adjusted *p*-values.

## 3. Results

### 3.1. Effects of Monascus pilosus SWM 008 Fermented Red Mold Rice and Its Compounds on Physiological and Biochemical Parameters in Rats

This study utilized the *Monascus pilosus* SWM 008 strain for large-scale fermentation of 200 kg of red mold rice. The fermentation products contained high amounts of monascin (MS), monascinol (Msol), and polysaccharides, all of which are functional yellow pigments known for their lipid-lowering effects and ability to improve metabolic syndrome. Given that Msol is a relatively lesser-studied yellow pigment, this study also aimed to investigate the safety of this strain and whether it has any regulatory effects on the gut microbiota of normal animals. This part of the experiment aimed to clarify whether *M. pilosus* SWM 008 and its yellow pigments and polysaccharides can improve related diseases through modulation of the gut microbiota.

Rats were fed a normal diet and administered *M. pilosus* SWM 008 fermented red mold rice, its functional polysaccharides, and yellow pigment solution via oral gavage for eight weeks. The effects on body weight were evaluated. As shown in Table 1, there were no significant differences in initial body weight, final body weight, or body weight gain across the different red mold rice test groups. Additionally, the various red mold test substances did not significantly affect liver and kidney weights. Table 1 also displays the effects of *M. pilosus* SWM 008 fermented red mold rice and its functional polysaccharides and yellow pigments on food intake and feed efficiency in rats. The results showed that red mold did not alter feed efficiency, caloric intake, or food consumption. These findings suggest that *M. pilosus* SWM 008 fermented red mold rice and its metabolites do not influence the feeding behavior of rats.

### 3.2. Effects of Monascus pilosus SWM 008 Fermented Red Mold Rice and Its Compounds on Serum Biochemical Parameters in Rats

To assess the effects of red mold test substances on liver function, serum levels of AST and ALT were measured. AST and ALT are important markers of liver function and reflect the extent of liver cell damage. The results in Table 2 indicate that the red mold test substances did not significantly alter AST and ALT levels, suggesting no adverse effects on liver function in rats on a normal diet. Additionally, as red mold has been shown to have lipid-lowering effects, serum cholesterol, and triglyceride levels were measured to determine whether the red mold test substances could regulate normal lipid metabolism. The results demonstrated no significant changes in serum cholesterol and triglyceride levels, indicating that the red mold test substances maintained normal lipid levels in rats on a regular diet.

Serum kidney function markers and coagulation factors were also measured to evaluate the effects of the red mold test substances on renal function and coagulation in rats on a normal diet. Kidney function markers included blood urea nitrogen (BUN) and uric acid, both of which are important indicators of the kidneys’ ability to excrete waste. Coagulation factors included CPK and coagulation factor II, both of which reflect blood coagulation status. As shown in Table 2, the red mold test substances did not significantly affect these parameters, suggesting no adverse effects on kidney function or coagulation in rats on a normal diet. Notably, Msol is a novel yellow pigment that was abundantly produced by the strain selected in this study, and prior experiments have demonstrated its lipid-lowering functionality. Thus, this functional yellow pigment Msol shows both efficacy and safety, as it does not elevate markers of liver or kidney function.

**Figure 1:**
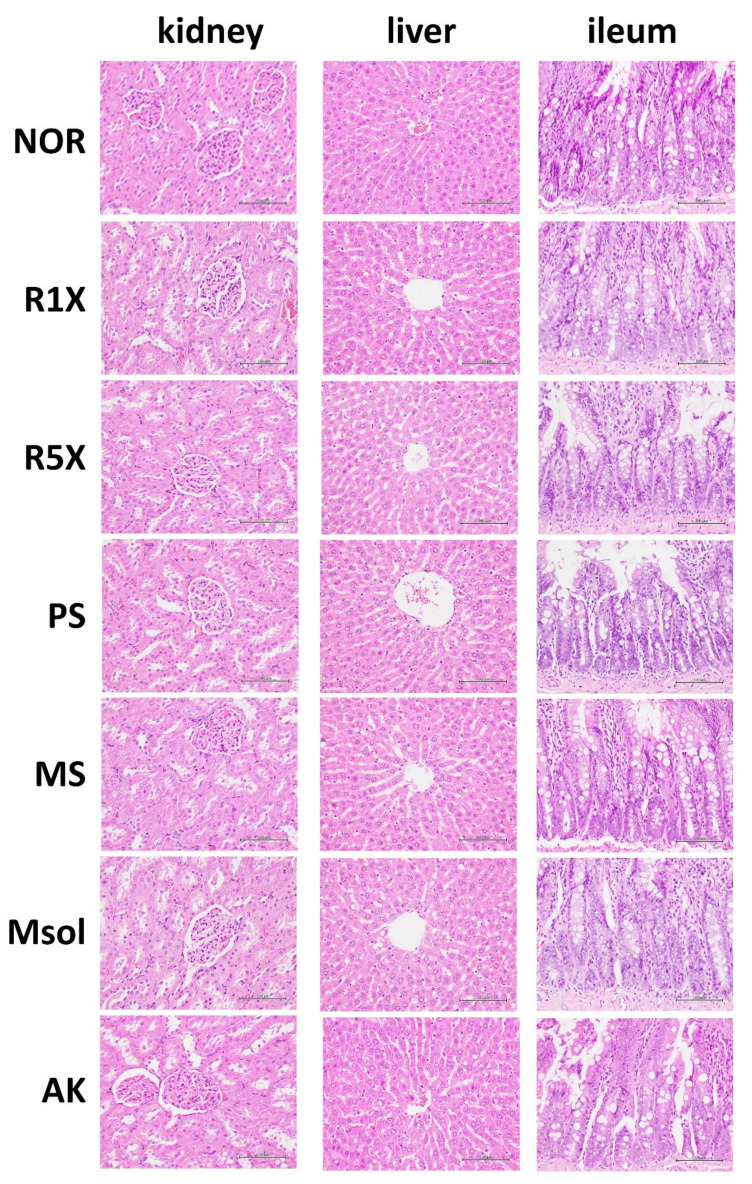
Hematoxylin and Eosin (H&E) Staining of Liver, Kidney, and Ileum in Rats Treated with *Monascus pilosus* SWM 008 Fermented Red Mold Rice and Its Components (400×).

Histopathological examinations were also conducted on the liver, kidney, and ileum of the experimental animals. As shown in Figure 1, after eight weeks of feeding with different test substances, no pathological damage was observed in the liver, kidneys, or ileum of the animals. This included findings similar to the NOR group, such as the absence of hepatic inflammation, renal edema or degeneration, and the maintenance of a generally intact and orderly arrangement of ileal villi. These results indicate the safety of the fermented red mold rice and its individual components.

### 3.3. High-Throughput Sequencing of Gut Microbiota Composition

**Figure 2:**
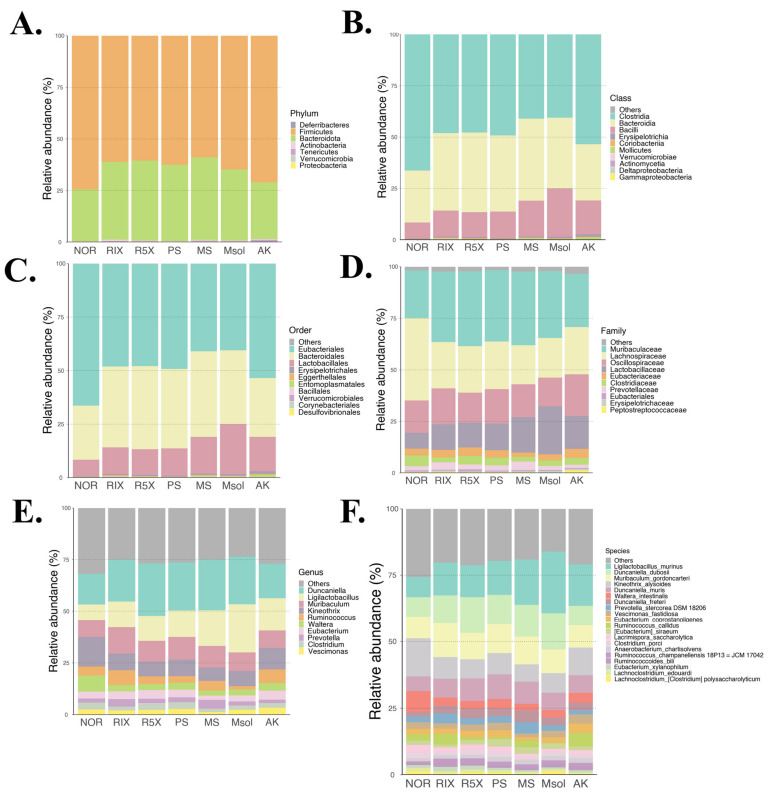
Comparative Analysis of the Top Microbial Taxa in the Gut Microbiota of Normal Diet Rats Treated with *Monascus pilosus* SWM 008 Fermented Red Mold Rice and Its Compounds: (**A**) Phylum, (**B**) Class, (**C**) Order, (**D**) Family, (**E**) Genus, and (**F**) Species Levels. Microbial abundance bar plots were generated from 16S rRNA sequencing data using DADA2, with taxonomic classifications assigned via reference databases. The top taxa at each level were visualized for group comparison.

Figure 2 presents the composition of gut microbiota at various taxonomic levels, including domain, class, order, family, genus, and species. At the phylum level, the data reveal that the gut microbiota is predominantly composed of Deferribacteres, Firmicutes, Bacteroidota, and Actinobacteria. These microbial phyla play diverse roles in gut function, especially in regulating energy metabolism and immune responses. Notably, Firmicutes and Bacteroidota have been strongly linked to obesity and metabolic diseases, with studies showing that shifts in the Firmicutes/Bacteroidota (F/B) ratio may affect host energy absorption efficiency [28].

At the class and order levels, Clostridia and Bacteroidia are the dominant taxa, contributing significantly to fiber fermentation, short-chain fatty acid production, and anti-inflammatory processes. The class Verrucomicrobiae holds particular importance for gut health, especially *Akkermansia muciniphila*, a known probiotic species that enhances gut barrier function and reduces obesity. At the family and genus levels, families such as Muribaculaceae, Lachnospiraceae, and Oscillospiraceae, and genera including *Ruminococcus*, *Prevotella*, and *Clostridium*, were identified as major contributors to host nutrient absorption, gut health, and disease prevention.

At the species level, *Ligilactobacillus murinus*, *Prevotella stercorea*, and *Eubacterium coprostanoligenes* are involved in crucial metabolic pathways, such as cholesterol metabolism, fatty acid breakdown, and immune regulation in the gut. According to the experimental results, treatment with fermented red mold rice and its functional components increased the relative abundance of species such as *Ligilactobacillus murinus*, *Duncaniella dubosii*, and *Muribaculum gordoncarteri*.

### 3.4. Alpha and Beta Diversity of Gut Microbiota

To assess the effect of red mold rice on gut microbiota, alpha diversity was analyzed using HiFi sequencing and the DADA2 algorithm to generate ASVs (Amplicon Sequence Variants). The alpha diversity, rarefaction curves, rank abundance curves, and heatmap clustering of abundance were evaluated across treatment groups.

**Figure 3:**
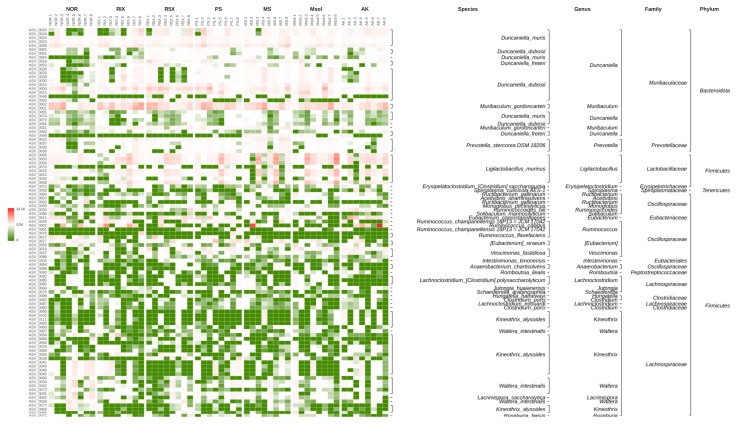
Heatmap of Relative Abundance and Taxonomic Annotation for Gut Microbial ASVs in Rats Treated with *Monascus pilosus* SWM 008 Fermented Red Mold Rice. The left side displays the relative abundance of the top 100 ASVs, with clustering based on abundance similarity. Taxonomic classification (Phylum, Family, Genus, Species) is shown on the right. The heatmap colors represent relative abundance values, with the median value used as the reference. ASV IDs are listed on the left, and different vertical blocks represent the treatment groups for comparison.

Figure 3 shows the relative abundance heat map of rat intestinal flora ASVs. It can be found that compared with the NOR group, each group fed the test substance increased the abundance of *Duncaniella*, showing potential anti-inflammatory effects [29]. It is worth mentioning that the Msol group has the effect of increasing the abundance of the probiotic *L. murinus* to increase rat metabolism and maintain intestinal health [30,31].

**Figure 4:**
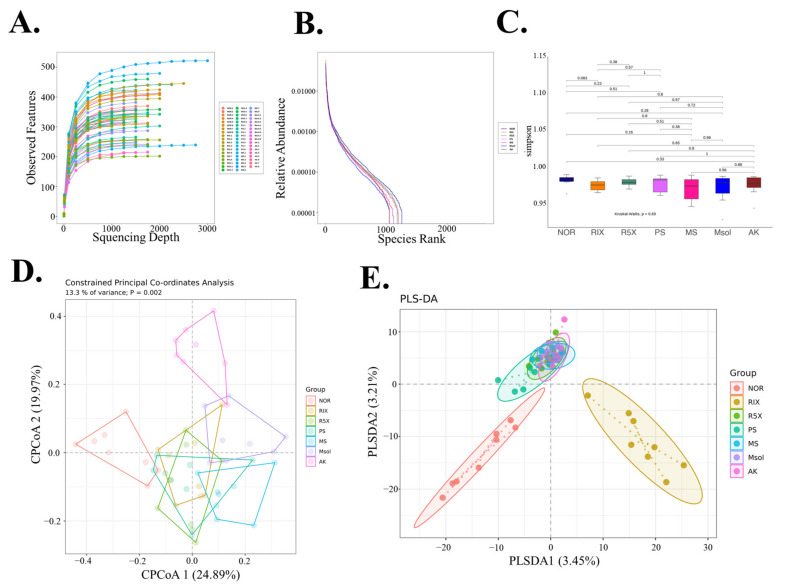
Alpha Diversity Analysis of Gut Microbiota in Rats Treated with *Monascus pilosus* SWM 008 Fermented Red Mold Rice. (**A**) Rarefaction Curves: X-axis shows the number of sampled sequences, and Y-axis indicates ASVs. Curves reflect sequencing depth and species richness. (**B**) Rank Abundance Curve: X-axis shows ASV rank by abundance, and Y-axis shows relative abundance. Curve width reflects species richness, and flatness shows evenness. (**C**) Alpha Diversity Boxplots: Boxplots show the distribution of alpha diversity indices (Observed Species and Shannon Index) for each group. Significance of group differences was tested using Kruskal–Wallis and Wilcoxon tests. (**D**) CPCoA Plot: Constrained Principal Coordinates Analysis shows group differentiation, with X and Y axes representing the first two components and their contribution to sample variation. (**E**) PLS-DA Plot: Partial Least Squares Discriminant Analysis illustrates group classification effectiveness, with each sample plotted based on its predicted group membership.

Figure 4A shows rarefaction curves illustrating ASV richness at various sequencing depths for each group. The x-axis represents sequencing depth, and the y-axis represents the observed ASVs. As sequencing depth increases, the curves plateau, indicating that sequencing depth was sufficient. The similar trends across groups suggest comparable richness in gut microbiota.

Figure 4B presents the rank abundance curves of different groups. The x-axis represents the ASV rank by abundance, and the y-axis indicates relative abundance. The shape of the curves reflects species richness and evenness, with minimal differences between the groups, indicating that red mold rice and its components had limited effects on microbial community structure.

The results in Figure 4C, based on Simpson’s diversity index, show no significant differences in alpha diversity between groups, as determined by the Wilcoxon paired test. This suggests that fermented red mold rice and its components had a limited effect on microbial diversity in the gut of rats, with stable gut microbiota diversity across different groups.

Figure 4D, the constrained principal coordinate analysis (CPCoA) plot, highlights the differences between groups. The NOR and AK groups are clearly separated on the CPCoA1 and CPCoA2 axes, reflecting significant differences in gut microbiota composition. R1X, R5X, PS, MS, and Msol are closely clustered, indicating similar effects on gut microbiota regulation.

Figure 4E illustrates the results of Partial Least Squares Discriminant Analysis (PLS-DA), showing clear distinctions between groups. The PLSDA1 and PLSDA2 axes indicate significant differences in gut microbiota composition between the NOR and R1X groups, with the NOR and R1X groups distinctly separated from other groups (R5X, PS, MS, Msol, and AK). In contrast, R5X, PS, MS, Msol, and AK showed similar gut microbial structures, implying similar regulatory effects on gut microbiota. Notably, lower doses (R1X) had more pronounced changes compared to higher doses (R5X), which showed consistent regulatory effects. These results suggest that fermented red mold rice and its functional components (PS, MS, Msol) have comparable effects on gut microbiota, while the NOR and AK groups display distinct microbial composition changes.

### 3.5. LEfSe and MetagenomeSeq Analysis of Gut Microbiota in Rats

**Figure 5:**
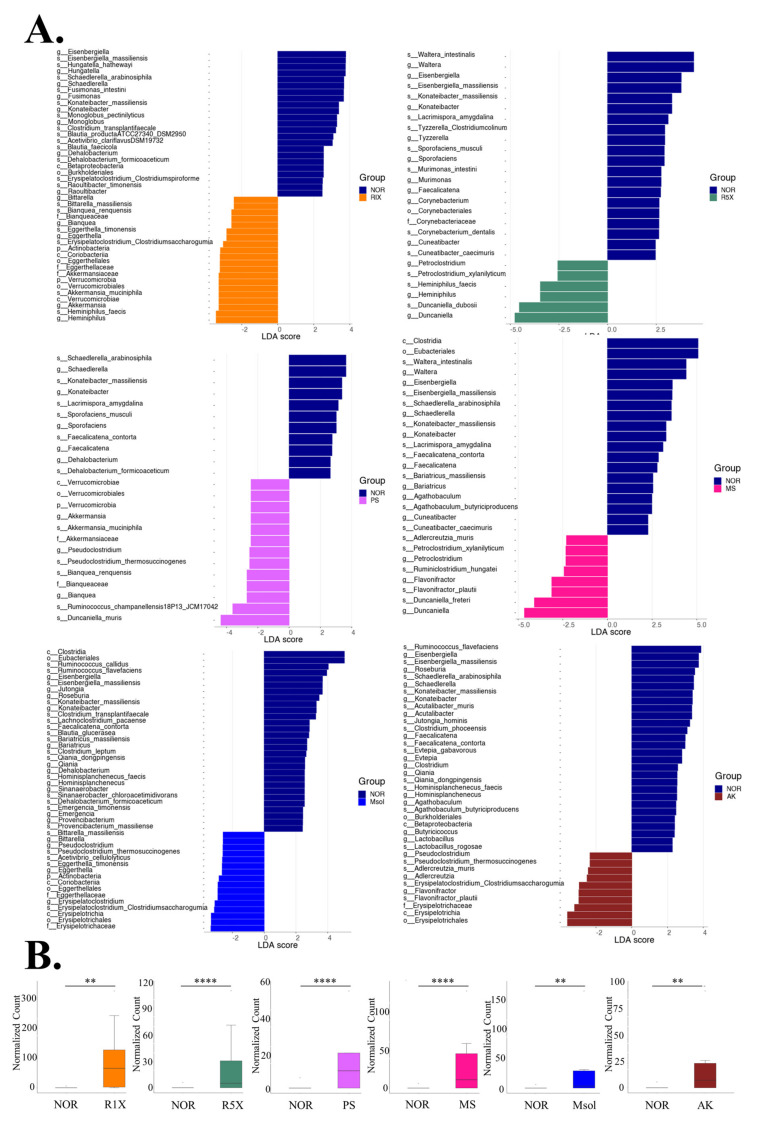
LEfSe and MetagenomeSeq Analysis of Gut Microbiota in Rats Treated with *Monascus pilosus* SWM 008 Fermented Red Mold Rice Compared to the NOR Group. (**A**) The LEfSe analysis identifies taxa with significant differences in abundance between the experimental groups and the NOR control group. The bar plot displays LDA scores (threshold set at 2.0), representing taxa with significant effects. The length of each bar indicates the impact size of the taxon, with colors representing different groups. The cladogram shows significant taxa across multiple taxonomic levels (Phylum to Species), with colored nodes corresponding to the group where the taxa are enriched. (**B**) MetagenomeSeq analysis focusing on the *Akkermansia* genus reveals significant differences in relative abundance across treatment groups. Using the zero-inflated log-normal model (fitZig), the analysis accounts for sequencing depth variations, with statistical significance determined using an FDR-adjusted *p*-value threshold of 0.05. ** indicated the significant difference *p* < 0.01, **** indicated the significant difference *p* < 0.0001. This plot highlights the genus-level differences in microbial abundance between groups.

Linear Discriminant Analysis (LDA) between the R1X and NOR groups revealed significant differences in gut microbiota composition (Figure 5A). The R1X group exhibited an increased abundance of *Bittarella massiliensis*, *Akkermansia muciniphila*, *Heminihilus faecis*, and *Eggerthella timonensis*. These taxa are associated with gut health and metabolic regulation, particularly *Akkermansia muciniphila*, which plays a critical role in enhancing gut barrier function and lipid metabolism. Conversely, the abundance of *Eisenbergiella massiliensis*, *Hungatella hathewayi*, and *Fusimonas intestini* decreased, suggesting that fermented red mold rice selectively suppresses potentially harmful bacteria, thereby enhancing metabolic health [32,33,34,35].

In the R5X group, compared to the NOR group, the abundance of Waltera intestinalis, Eisenbergiella massiliensis, Konateibacter massiliensis, and Murimonas intestini decreased, while Petroclostridium xylanilyticum, Heminihilus faecis, and Duncaniella dubosii showed an increase. This suggests that higher doses of fermented red mold rice selectively promote beneficial bacteria while reducing others. The PS (polysaccharide) group also exhibited notable changes. Schaedlerella arabinosiphila, Konateibacter massiliensis, and Lacrimispora amygdalina decreased, whereas Akkermansia muciniphila, Pseudoclostridium thermosuccinogenes, and Ruminococcus champanellensis increased. These findings imply that red mold polysaccharides promote the growth of beneficial bacteria linked to enhanced gut and metabolic health. In the MS (monascin) group, there was a significant reduction in Clostridia, Eubacteriales, Waltera intestinalis, and Eisenbergiella massiliensis, while Adlercreutzia muris, Petroclostridium xylanilyticum, and Duncaniella freteri showed an increase. These results indicate that monascin fosters beneficial taxa, potentially influencing metabolic and immune responses. The Msol (monascinol) group demonstrated a decrease in Clostridia, Ruminococcus callidus, and Eisenbergiella massiliensis, while Bittarella massiliensis, Pseudoclostridium thermosuccinogenes, and Eggerthella timonensis increased. These observations suggest that monascinol modulates the gut microbiota with positive implications for metabolic health and immune regulation. In the AK (ankaflavin) group, reductions in Ruminococcus flavefaciens, Eisenbergiella massiliensis, and Roseburia were noted, whereas Pseudoclostridium thermosuccinogenes, Adlercreutzia muris, and Flavonifractor plautii showed increased abundance. This indicates that ankaflavin modulates the composition of the gut microbiota to potentially regulate immune responses and metabolic pathways that contribute to improved gut health. As show in Figure 5B MetagenomeSeq analysis for the genus Akkermansia, our results demonstrate that all tested substances effectively increase the abundance of this probiotic.

### 3.6. PICRUSt2 Functional Prediction of Gut Microbiota in Rats

**Figure 6:**
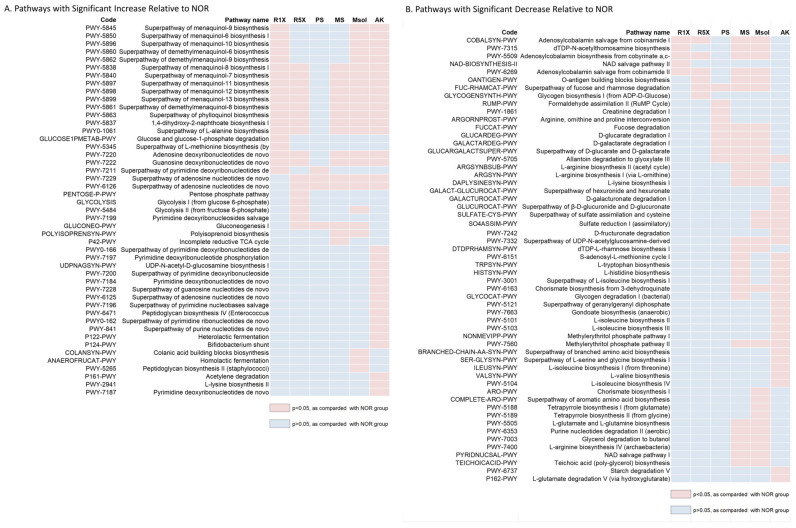
PICRUSt2 Functional Prediction of Gut Microbiota in Rats Treated with *Monascus pilosus* SWM 008 Fermented Red Mold Rice. (**A**) Functional pathways significantly increased across treatment groups compared to NOR. (**B**) Functional pathways significantly decreased across treatment groups compared to NOR. Red squares indicate significant differences (*p* < 0.05, determined by Welch’s *t*-test with Bonferroni correction), while blue squares represent non-significant changes.

We conducted a functional prediction analysis of metabolic pathways in the experimental groups (R1X, R5X, PS, MS, Msol, AK) compared to the control group (NOR) (Figure 6). Significant differences were observed, which may have important implications for host health and microbiota function.

Vitamin K2 biosynthesis pathways, such as PWY-5838 (menaquinol-8 biosynthesis), were significantly upregulated in the R1X, R5X, MS, Msol, and AK groups, potentially supporting bone health and blood coagulation.

Red mold rice, MS, Msol, and AK demonstrated increased nucleotide synthesis, with significant upregulation of pathways like PWY-7220 (adenosine deoxyribonucleotides de novo biosynthesis II). This suggests enhanced DNA and RNA synthesis capacity, which supports cell proliferation and immune function. Amino acid metabolism showed significant upregulation in the PWY0-1061 (L-alanine biosynthesis) pathway across experimental groups (R1X, R5X, MS, Msol, AK), indicating increased amino acid biosynthesis to support protein synthesis and energy metabolism. Additionally, gluconeogenesis (GLUCONEO-PWY) was significantly upregulated in the PS, MS, and Msol groups, contributing to stable blood glucose levels and energy supply.

Conversely, vitamin B12 metabolism pathways (COBALSYN-PWY, PWY-5509) were downregulated in the R1X, R5X, MS, and Msol groups, potentially reducing B12 synthesis and impacting energy metabolism and neurological functions. Carbohydrate degradation pathways (FUC-RHAMCAT-PWY, GLUCARDEG-PWY) were significantly downregulated in the PS, MS, and Msol groups, potentially affecting the breakdown of specific carbohydrates and the production of short-chain fatty acids.

The MS and Msol groups showed reduced L-arginine biosynthesis (ARGSYNBSUB-PWY, ARGSYN-PWY), which could affect protein metabolism and nitric oxide production. The AK group also exhibited a decrease in the BRANCHED-CHAIN-AA-SYN-PWY (branched-chain amino acid biosynthesis), which may impact muscle protein metabolism and energy production.

Overall, the upregulation of vitamin K2 and nucleotide metabolism across most groups suggests enhanced microbial activity and potential benefits for host health. However, the downregulation of vitamin B12 metabolism, carbohydrate degradation, and amino acid biosynthesis may negatively affect nutrient absorption and metabolic balance. These findings offer insights into the interplay between gut microbiota and host health, providing valuable references for future clinical applications.

### 3.7. Correlation Analysis and Redundancy Analysis (RDA) of Gut Microbiota in Rats

**Figure 7:**
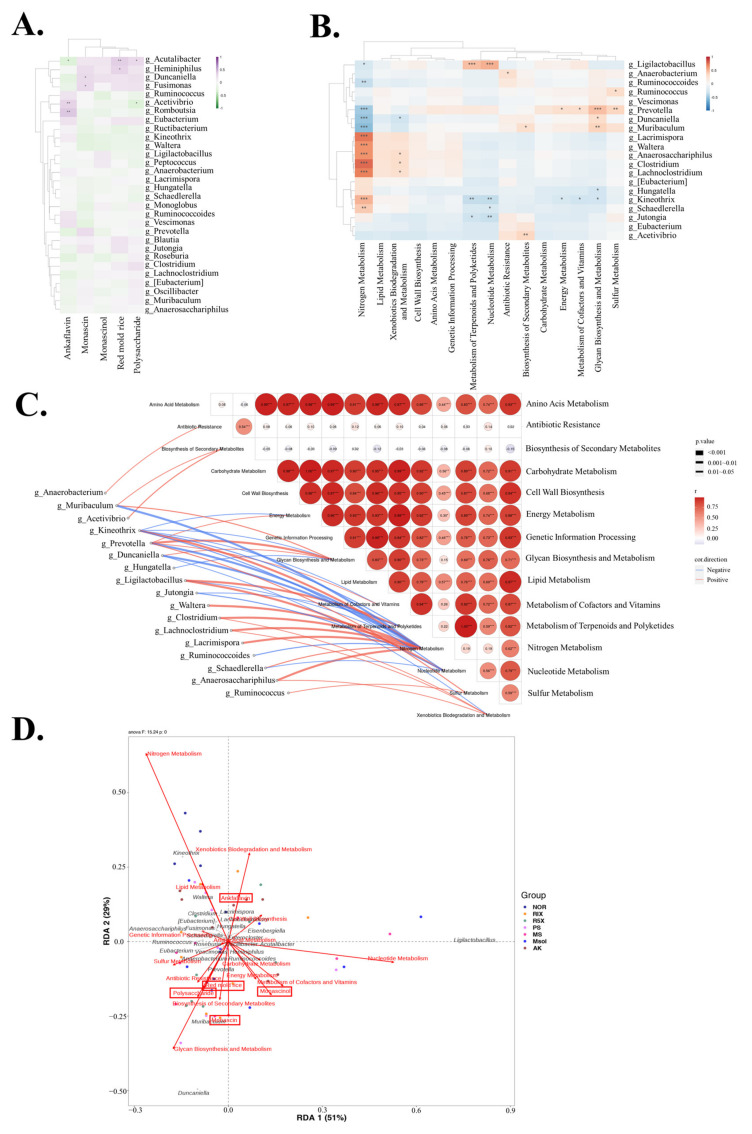
Correlation Analysis and Redundancy Analysis (RDA) of Gut Microbiota in Rats Treated with *Monascus pilosus* SWM 008 Fermented Red Mold Rice. (**A**) Heatmap displaying the correlation between the top 20 genera and predicted metabolic pathways. (**B**) Heatmap showing the correlation between the top 20 genera and experimental factors. (**C**) Correlogram illustrating genus-function relationships, with red and blue lines indicating positive and negative correlations, respectively. (**D**) Constrained Ordination Plot using Redundancy Analysis (RDA), integrating the top 20 genera, predicted metabolic pathways, and experimental factors. Pearson correlation and FDR-adjusted *p*-values were applied to assess statistical significance. * indicated the significant difference *p* < 0.05, ** indicated the significant difference *p* < 0.01, *** indicated the significant difference *p* < 0.001.

Correlation analysis was performed to evaluate the relationships between the top 30 genera and the various treatments (red mold rice, polysaccharides, monascin, monascinol, and ankaflavin). The results (Figure 7A) demonstrate that each treatment induced distinct shifts in gut microbiota composition. For the red mold rice treatment, *Heminiphilus*, *Acutalibacter*, and *Fusimonas* showed increased abundance, indicating a positive response. In the polysaccharide group, *Duncaniella*, *Fusimonas*, and *Acutalibacter* increased, while *Acetivibrio* decreased. Administration of monascin led to increases in *Duncaniella*, *Prevotella*, and *Fusimonas*, while *Kineothrix* decreased. For monascinol, *Duncaniella* and *Acutalibacter* increased, whereas *Ruminococcus* and *Acetivibrio* decreased. Ankaflavin treatment resulted in an increase in *Acetivibrio* and *Ruminococcus*, while *Acutalibacter* decreased. These treatments selectively influenced genera such as *Heminiphilus*, *Duncaniella*, and *Acutalibacter*, indicating targeted effects on the gut microbiota.

Correlation analysis between bacterial genera and metabolic pathways (Figure 7B,C) revealed distinct functional relationships. *Muribaculum* was positively correlated with vitamin metabolism and xenobiotic biodegradation, suggesting a role in nutrient absorption and detoxification. *Prevotella* was positively correlated with vitamin and carbohydrate metabolism, indicating its involvement in nutrient utilization. *Clostridium* and *Lachnoclostridium* were linked to nucleotide metabolism, highlighting their roles in cell proliferation and energy supply. Conversely, Duncaniella was negatively correlated with nucleotide and xenobiotic metabolism, suggesting limited activity in these pathways. Positive correlations observed in genera like *Muribaculum* and *Prevotella* underscore their significance in maintaining host nutritional balance, while negative correlations indicate lower participation in certain metabolic functions.

The redundancy analysis (RDA) plot (Figure 7D) illustrates relationships between bacterial genera and metabolic pathways, with points divided into four quadrants. Red mold rice and its components (monascin, monascinol, and polysaccharides) showed similar directional trends, whereas ankaflavin exhibited an opposing trend. *Ruminococcus* correlated positively with sulfur metabolism, suggesting involvement in sulfur compound reduction. *Muribaculum*, *Heminiphilus*, and *Duncaniella* aligned with glycan biosynthesis, secondary metabolite biosynthesis, and antibiotic resistance pathways, implying roles in carbohydrate synthesis, metabolite production, and host defense. Monascinol was associated with *Ruminococcoides*, which was positively correlated with energy and vitamin metabolism. *Vescimonas* was linked to carbohydrate and energy metabolism, while *Ligilactobacillus* correlated with nucleotide and terpenoid/polyketide metabolism, though no treatment was strongly linked to *Ligilactobacillus*. Ankaflavin correlated with *Lachnoclostridium* and *Lacrimispora*, which were linked to xenobiotic biodegradation, lipid metabolism, and cell wall biosynthesis. These results underscore the diverse roles of different genera in host metabolic processes, particularly energy production, vitamin synthesis, and defense mechanisms.

## 4. Discussion

Recent studies highlight the benefits of monascin and ankaflavin, compounds derived from *Monascus*, in reducing body fat and improving cardiovascular health. Chen et al. (2017) found that Ankascin 568, rich in these compounds, significantly reduced blood pressure and improved lipid profiles, including lower total cholesterol (TC), reduced LDL-C, and increased HDL-C [1]. Liu et al. (2018) also reported reductions in TC and LDL-C with Ankascin 568 Plus, supporting its role in lipid regulation [2]. A review by Chen and Pan confirmed these cholesterol-lowering effects, suggesting a potential for cardiovascular health management [36].

More recent research has highlighted the hypolipidemic properties of monascinol, including its ability to decrease the expression of fatty acid synthase (FASN) and sterol regulatory element-binding protein 2 (SREBP2), and to promote triglyceride and bile acid excretion. Notably, monascinol has demonstrated greater efficacy than monascin in enhancing AMPK phosphorylation and reducing liver inflammation in hyperlipidemic models [3]. Together, monascin, monascinol, and ankaflavin have demonstrated their potential to mitigate cardiovascular and metabolic syndrome-related complications, particularly in the context of high-fat diets. Studies have shown that red mold rice can effectively reduce lipid levels, inhibit hepatic lipid accumulation, and enhance cholesterol and bile acid excretion while simultaneously modulating gut microbiota to counteract high-fat diet-induced dysbiosis. For example, red mold rice significantly reduced cholesterol, LDL, and arterial plaque through TLR2 and TLR4 pathways, while monascin alleviated lipid metabolism disorders by modulating key gene expressions and enhancing beneficial bacterial populations [5,6]. However, the majority of existing research has focused on high-fat diet models, leaving uncertainties regarding red mold rice’s modulation of microbiota in standard dietary contexts. This study addresses this gap by utilizing a non-high-fat diet model to evaluate red mold rice and its pigments for their prebiotic potential and modulation of gut microbiota. The results suggest that red mold rice enhances microbiome diversity and increases beneficial bacterial populations, thereby supporting its use as a functional food for metabolic health and microbiota modulation.

This study employed high-throughput sequencing to analyze gut microbiota composition in rats, revealing significant shifts in the abundance of various phyla and genera in response to red mold rice and its functional components—polysaccharides, monascin, monascinol, and ankaflavin. Several taxa closely associated with host metabolism and immune regulation, such as *Firmicutes*, *Bacteroidota*, *Akkermansia muciniphila*, and *Ligilactobacillus murinus*, exhibited altered abundance. These bacteria are believed to play crucial roles in energy metabolism, short-chain fatty acid (SCFA) production, and immune modulation.

The Firmicutes/Bacteroidota ratio is associated with metabolic health, with an elevated ratio linked to obesity, inflammation, and metabolic disorders, particularly under high-fat dietary conditions [28,37]. Bacteroidota, on the other hand, enhance energy metabolism by fermenting dietary fibers into SCFAs with anti-inflammatory properties [38]. *Akkermansia muciniphila* and *Ligilactobacillus murinus* play critical roles in gut health; the former strengthens the gut barrier, reduces inflammation, and improves metabolic outcomes in obesity and diabetes [4,39], while the latter stabilizes gut microbial communities and promotes gut health [40]. In this study, the intervention with red mold rice and its components did not affect the F/B ratio However, the relative abundance of many beneficial bacteria significantly increased, which may have a positive impact on gut health and metabolic regulation.

This study assessed changes in gut microbiota through both alpha and beta diversity analyses. Alpha diversity, evaluated using the Simpson diversity index, measures microbial richness and evenness, which are critical indicators of gut health. Increased alpha diversity generally reflects a more balanced and resilient microbial community, which is associated with improved gut health [41]. Beta diversity, used to compare differences in microbial composition across groups, was assessed using Principal Coordinates Analysis (PCoA) and Partial Least Squares-Discriminant Analysis (PLS-DA). These methods illustrated shifts in microbial community structure between the different groups, highlighting the impact of specific dietary or therapeutic interventions. Previous studies employing PLS-DA have shown significant variations in gut microbiota structure among intervention groups, emphasizing the influence of diet or treatment on microbial composition [42]. In this study, PLS-DA results indicated a clear distinction between the NOR and R1X groups, whereas the R5X, PS, MS, Msol, and AK groups exhibited similar microbiota structures, suggesting that higher doses of red mold rice and its components exert a consistent regulatory effect on gut microbiota. The CPCoA analysis further supported this, showing distinct gut microbiota structures in the NOR and AK groups, while other functional component groups exhibited similar regulatory patterns. Different doses and components of red mold rice demonstrated pronounced effects on gut microbiota, with more significant changes observed at lower doses (R1X) and a more consistent regulatory effect at higher doses (R5X).

Based on LEfSe and MetagenomeSeq analyses, *Monascus pilosus* SWM 008-fermented red mold rice and its functional components significantly modulated gut microbiota composition across treatment groups, particularly by increasing beneficial bacteria such as *Bittarella massiliensis*, *Akkermansia muciniphila*, and *Heminiphilus faecis*. Some of these bacteria have potential health benefits for the host. [43,44]. *Akkermansia muciniphila* enhances gut integrity, regulates lipids, and improves obesity and diabetes outcomes [45,46]. These findings suggest that functional components of red mold rice, including monascin, monascinol, and polysaccharides, contribute to gut health by fostering the growth of beneficial bacteria. In the R1X group, *Akkermansia muciniphila* showed significant increases compared to the NOR group, underscoring its role in enhancing gut barrier function and promoting lipid metabolism [4]. *Akkermansia muciniphila* also secretes P9 protein, which has been shown to boost GLP-1 secretion, thereby improving glucose metabolism [45]. The intervention with red mold rice also selectively reduced potentially harmful bacteria such as *Eisenbergiella massiliensis* and *Hungatella hathewayi*, indicating targeted modulation of the gut microbiota. In the R5X group, a higher dose of red mold rice led to significant shifts in gut microbiota, with a reduction in *Waltera intestinalis* and *Konateibacter massiliensis* and an increase in *Petroclostridium xylanilyticum* and *Duncaniella dubosii*, indicating a dose-dependent effect. The PS group showed an increase in bacteria linked to gut barrier function and metabolism, such as *Akkermansia muciniphila* and *Ruminococcus champanellensis*, suggesting that polysaccharides promote gut health and SCFA production [47]. Both monascin and monascinol significantly enhanced *Akkermansia muciniphila* levels, highlighting their potential to improve metabolic health by supporting beneficial bacteria. Monascinol also increased *Adlercreutzia muris* and *Petroclostridium xylanilyticum*, whereas monascinol boosted *Bittarella massiliensis* and *Pseudoclostridium thermosuccinogenes*, reinforcing their roles in gut microbiota regulation and metabolic benefits [48]. Overall, red mold rice and its components, including monascin and monascinol, modulate the gut microbiota by promoting beneficial bacteria such as *Akkermansia muciniphila* and reducing harmful bacteria, contributing to improved gut and metabolic health.

Based on PICRUSt2 analysis, this study revealed significant metabolic pathway changes across experimental groups, underscoring their potential impact on host health and gut microbiota metabolism. Specifically, pathways related to the biosynthesis of vitamin K2 (menaquinone) were upregulated in the R1X, R5X, MS, Msol, and AK groups. Vitamin K2, synthesized by gut microbes, plays an essential role in blood coagulation and bone health, with various forms of menaquinone (MK) linked to improved bone density and cardiovascular health [49,50]. These findings suggest that fermented red mold rice and its functional components enhance vitamin K2 production, contributing to improved host metabolic health. Additionally, nucleotide synthesis pathways were upregulated across all experimental groups, indicating an enhanced capacity for DNA and RNA synthesis, which supports cell proliferation and immune function [51]. Enhanced amino acid metabolism, specifically the superpathway for L-alanine biosynthesis, was also observed across all groups, suggesting increased protein synthesis and energy metabolism by gut microbiota. The upregulation of gluconeogenesis pathways in the PS, MS, and Msol groups may help maintain stable blood glucose levels and energy supply, which are crucial for metabolic health [52]. However, pathways related to vitamin B12 synthesis were downregulated in the R1X and R5X groups, suggesting a potential decrease in vitamin B12 production, which may affect energy metabolism and neurological health [53]. Overall, the study highlights the broad metabolic effects of fermented red mold rice and its components, particularly the upregulation of vitamin K2 and nucleotide metabolism, which may enhance host metabolic health. These findings provide important insights for future research into the clinical potential of fermented red mold rice.

According to the literature, red mold rice and its functional components, such as polysaccharides, monascin, monascinol, and ankaflavin, significantly influence gut microbiota and are linked to the expression of functional genes. Previous studies have shown that polysaccharides increase the abundance of beneficial bacteria such as *Duncaniella* and *Fusimonas*, which are associated with vitamin and carbohydrate metabolism, promoting host energy balance and metabolic health [54,55]. Monascin and monascinol, as functional pigments, primarily affect nucleotide and vitamin synthesis pathways, promoting cell proliferation and energy metabolism. Further literature indicates that gut bacteria with anti-inflammatory and immune-regulatory properties, such as *Akkermansia*, significantly increased following red mold rice intervention (including polysaccharides, monascin, and monascinol), contributing to improved fat metabolism and immune function [56]. These bacteria play a crucial role in carbohydrate degradation, SCFA production, and glycogen storage, which are vital for maintaining energy metabolism and immune function [57]. Additionally, the growth of *Prevotella*, *Duncaniella*, and *Muribaculum* correlated with the upregulation of vitamin K2 biosynthesis pathways [58], suggesting that red mold rice components promote bone health and support blood clotting functions.

This study demonstrates that red mold rice and its components (such as monascin, monascinol, and polysaccharides) positively modulate gut microbiota even under normal dietary conditions. This is the first evidence that these components can improve gut microbiota composition without the induction of a high-fat diet. The key components of red mold rice were found to be significantly positively correlated with specific gut bacterial genera and metabolic pathways, particularly those related to carbohydrate synthesis, secondary metabolite production, and antibiotic resistance. These correlations suggest that certain bacterial genera may play a critical role in host metabolic regulation and defense mechanisms. Specifically, redundancy analysis (RDA) revealed positive associations between red mold rice components such as monascin and polysaccharides with bacteria like *Muribaculum* and *Duncaniella*, suggesting that these components may enhance host metabolic function by influencing carbohydrate and secondary metabolite synthesis pathways, with a particular emphasis on anti-inflammatory and antioxidant activities [59].

Furthermore, the positive correlation between *Ruminococcus* and sulfur metabolism suggests that red mold rice may contribute to sulfur compound reduction or sulfate metabolism, functions closely tied to maintaining microbial diversity and gut health, potentially reducing oxidative stress and improving outcomes in hyperlipidemia [60]. In contrast, ankaflavin was associated with bacterial genera involved in lipid metabolism, xenobiotic degradation, and cell wall synthesis, implying its role in lipid synthesis and breakdown, as well as in clearing harmful substances and maintaining metabolic balance and cellular integrity [61]. Based on the study results, red mold rice and monascinol showed strong positive correlations with the biosynthesis of vitamin K2 (menaquinone) pathways, suggesting their role in promoting vitamin K2 production by gut microbiota. Vitamin K2 is critical for cardiovascular health, particularly in reducing arterial calcification and improving blood clotting by promoting calcium regulation. This correlation highlights the potential role of red mold rice in supporting cardiovascular health through vitamin K2 biosynthesis, aligning with previous studies linking vitamin K2 to cardiovascular protection [62,63]. The experiment also identified positive correlations between menaquinone biosynthesis and specific gut bacteria, particularly *Prevotella* and *Muribaculum*, which are involved in metabolic pathways such as carbohydrate metabolism and SCFA synthesis, both vital for host metabolic health [64]. Increased menaquinone production by these bacteria not only supports bone health and reduces arterial stiffness but also contributes to overall cardiovascular protection by preventing vascular calcification, suggesting that gut microbiota plays a crucial role in the cardiovascular benefits of red mold rice [62,65].

This study is the first to confirm that red mold rice and its functional components, such as polysaccharides, monascin, and monascinol (yellow pigments), significantly regulate gut microbiota not only in animal models subjected to high-fat diets but also under normal dietary conditions. This finding broadens the understanding of the mechanisms underlying red mold rice’s action, highlighting its potential to enhance gut health without reliance on a high-fat diet. Previous research primarily focused on the ability of red mold rice to modulate gut microbiota in the context of high-fat diets and its association with improvements in metabolic disorders, such as obesity, diabetes, and dyslipidemia. For instance, gut microbiota imbalance caused by a high-fat diet has been shown to significantly alter overall microbial structure following supplementation with *Monascus* pigments. Unlike previous reports that indicated a decrease in *Clostridium* abundance due to a high-fat diet and a subsequent significant increase following *Monascus* pigment supplementation, this study found that under a normal diet, *Monascus* pigments did not significantly increase *Clostridium* abundance [6]. However, the results of this study indicate that red mold rice and its components, unlike the effects observed with a high-fat diet, can promote gut microbial diversity and stability even under normal dietary conditions, demonstrating broad health benefits.

The regulatory effects of polysaccharides and yellow pigments in red mold rice may be closely related to their antioxidant, anti-inflammatory, and lipid-lowering properties. High-fat diets often induce gut microbiota imbalance, leading to chronic inflammation, insulin resistance, and dyslipidemia. Studies have shown that high-fat diets increase harmful bacteria such as *Firmicutes* while reducing beneficial bacteria like *Akkermansia* and *Bacteroidetes* [66]. Red mold rice and its functional components counteract this dysbiosis by promoting the growth of beneficial bacteria and reducing the abundance of harmful bacteria. This study’s results show that even under normal dietary conditions, red mold rice and its components effectively increased the abundance of beneficial bacteria such as *Akkermansia*, helping maintain gut barrier integrity, reduce intestinal permeability, endotoxemia, and chronic inflammation—all of which are closely linked to obesity and metabolic syndrome [67].

## 5. Conclusions

This study demonstrated that *Monascus pilosus* SWM 008 fermented red mold rice and its key components—monascin, monascinol, ankaflavin, and polysaccharides—are safe and effective in modulating gut microbiota composition in rats fed a normal diet. Over the eight-week study period, no adverse effects on physiological or biochemical parameters, including liver and kidney function, were observed, confirming the safety of these compounds. The fermented red mold rice and its functional components significantly altered gut microbiota, increasing the abundance of beneficial bacteria such as *Akkermansia muciniphila*, *Ligilactobacillus murinus*, and *Duncaniella dubosii*. These changes were associated with enhanced metabolic functions, including increased vitamin K2 biosynthesis, nucleotide metabolism, and amino acid biosynthesis. Conversely, certain metabolic pathways, such as vitamin B12 metabolism and carbohydrate degradation, were downregulated, which may impact nutrient absorption and metabolic balance.

The findings suggest that fermented red mold rice and its components hold promise as functional foods for promoting metabolic health and maintaining gut microbiota diversity, even under normal dietary conditions. The potential of *Monascus*-fermented products as dietary interventions for metabolic disorders, such as obesity and dyslipidemia, warrants further investigation. Although this experiment did not include a high-fat diet comparison, we observed differences in gut microbiota changes between normal diet-fed red mold rice and those reported in previous studies using high-fat diet-fed red mold rice. It remains unclear whether these differences are due to strain-specific effects or dietary interventions. Nevertheless, this study demonstrates that pure red mold rice and its components possess the ability to enhance beneficial microbial species and associated metabolic pathways, supporting their potential health-promoting properties.

## Figures and Tables

**Table 1:** Effects of *Monascus pilosus* SWM 008 Fermented Red Mold Rice and Its Compounds on Body Weight, Food Intake, and Organ Weights in Rats.

Groups	NOR	R1X	R5X	PS	MS	AK	Msol
Food intake (g)	1479.2 ± 71.2	1469.9 ± 81.9	1488.1 ± 81.6	1490.0 ± 55.1	1527.9 ± 36.7	1531.6 ± 58.4	1526.7 ± 50.8
Calorie intake (kcal)	4961.2 ± 238.7	4930.2 ± 274.8	4991.0 ± 273.6	4997.5 ± 184.8	5073.6 ± 324	4992.7 ± 322.7	5035.1 ± 328.4
Feed efficiency (%)	16.5 ± 0.8	16.8 ± 1.0	16.2 ± 1.7	16.0 ± 1.1	16.4 ± 0.9	16.5 ± 1.0	16.5 ± 0.9
Initial body weight (g)	249.5 ± 5.8	245.9 ± 6.3	251.5 ± 7.8	249.8 ± 5.8	248.6 ± 6.7	254.7 ± 7.4	253.3 ± 7.1
Final body weight (g)	493.8 ± 19.3	493.4 ± 26	492.1 ± 26.6	488.1 ± 16.9	495.7 ± 19.2	512.5 ± 27.6	512.7 ± 21.2
body weight gain (g)	244.3 ± 17.6	247.5 ± 20.9	240.6 ± 27.7	238.4 ± 16.4	247.1 ± 15.2	257.8 ± 25	259.5 ± 17.5
liver weight (g)	13.56 ± 0.90	13.30 ± 1.05	13.36 ± 0.59	13.01 ± 0.54	14.15 ± 0.75	13.84 ± 0.59	14.44 ± 0.79
liver weight/body weight (%)	2.74 ± 0.13	2.67 ± 0.11	2.72 ± 0.09	2.67 ± 0.11	2.78 ± 0.18	2.73 ± 0.20	2.74 ± 0.12
kidney weight (g)	3.59 ± 0.16	3.60 ± 0.20	3.56 ± 0.17	3.58 ± 0.29	3.54 ± 0.12	3.55 ± 0.22	3.53 ± 0.16

There are no restrictions on food and water intake in all groups. NOR: This group serves as the negative control, receiving only RO water, R1X: administered *Monascus pilosus* SWM 008-fermented red mold rice 0.103 g/kg b.w. rat/day, R5X: administered *Monascus pilosus* SWM 008-fermented red mold rice 0.517 g/kg b.w. rat/day, PS: administered *Monascus* polysaccharide 0.021 g/kg b.w. rat/day, MS: administered monascin 0.31 mg/kg b.w. rat/day, AK: administered ankaflavin 0.31 mg/kg b.w. rat/day, Msol: administered monascinol 0.31 mg/kg b.w. rat/day. Data are presented as means ± SD (*n* = 8). Mean values with different letters are significantly different (*p* < 0.05). The statistical significance in the biochemical effects was determined by ANOVA with Duncan’s multiple-range test. No statistical differences were observed in the data presented in this table; therefore, statistical markers are not indicated.

**Table 2:** Effects of *Monascus pilosus* SWM 008 Fermented Red Mold Rice and Its Compounds on Serum Biochemical Parameters in Rats.

Group	NOR	R1X	R5X	PS	MS	AK	Msol
TG (mg/dL)	72.00 ± 20.61	83.00 ± 10.54	74.25 ± 18.50	77.38 ± 17.93	81.13 ± 8.25	82.00 ± 19.8	80.25 ± 16.52
TC (mg/dL)	79.00 ± 11.00	80.25 ± 7.74	75.38 ± 14.19	73.13 ± 4.78	72.88 ± 6.47	77.88 ± 11.15	75.25 ± 10.66
AST (U/L)	106.00 ± 10.11	102.75 ± 8.15	102.88 ± 10.78	107.38 ± 15.26	92.88 ± 5.71	99.75 ± 9.58	96.75 ± 8.30
ALT (U/L)	49.50 ± 6.00	47.75 ± 4.66	47.25 ± 7.24	47.13 ± 6.33	42.50 ± 6.34	43.00 ± 7.00	43.50 ± 3.91
BUN (mg/dL)	14.36 ± 0.85	13.44 ± 0.54	14.29 ± 0.98	13.80 ± 0.69	14.94 ± 0.45	14.73 ± 0.79	14.29 ± 0.48
Uric acid (mg/dL)	6.29 ± 0.88	6.06 ± 0.52	6.10 ± 0.62	5.88 ± 0.61	5.56 ± 0.37	5.58 ± 0.57	5.36 ± 0.48
CPK (U/L)	280.75 ± 92.19	234.25 ± 40.97	288.75 ± 72.79	308.38 ± 75.00	286.75 ± 32.26	258.00 ± 55.81	248.25 ± 36.01
Coagulation Factor II (mg/L)	36.75 ± 3.87	38.86 ± 8.78	38.57 ± 8.48	38.49 ± 6.27	37.63 ± 6.53	37.33 ± 7.65	37.35 ± 7.34

There are no restrictions on food and water intake in all groups. NOR: This group serves as the negative control, receiving only RO water, R1X: administered *Monascus pilosus* SWM 008-fermented red mold rice 0.103 g/kg b.w. rat/day, R5X: administered *Monascus pilosus* SWM 008-fermented red mold rice 0.517 g/kg b.w. rat/day, PS: administered *Monascus* polysaccharide 0.021 g/kg b.w. rat/day, MS: administered monascin 0.31 mg/kg b.w. rat/day, AK: administered ankaflavin 0.31 mg/kg b.w. rat/day, Msol: administered monascinol 0.31 mg/kg b.w. rat/day. Data are presented as means ± SD (*n* = 8). Mean values with different letters are significantly different (*p* < 0.05). The statistical significance in the biochemical effects was determined by ANOVA with Duncan’s multiple-range test. No statistical differences were observed in the data presented in this table; therefore, statistical markers are not indicated. TG: triglyceride, TC: total cholesterol, AST: aspartate transaminase, ALT: alanine transaminase, BUN: blood urea nitrogen, CPK: creatine phosphokinase.

## Data Availability

The original contributions presented in this study are included in the article/Supplementary Materials. Further inquiries can be directed to the corresponding authors.

## Supplementary Materials

The following supporting information can be downloaded at: https://www.mdpi.com/article/10.3390/foods14040651/s1, Table S1: Test substance ankaflavin certificate of analysis. Table S2: Test substance monascin certificate of analysis. Table S3: Test substance monascinol certificate of analysis. Figure S1: HPLC analysis of monascin. Figure S2: HPLC analysis of ankaflavin. Figure S3: HPLC analysis of monascinol.
