# Supplementary material for: Effects of Monascus pilosus SWM 008-Fermented Red Mold Rice and Its Functional Components on Gut Microbiota and Metabolic Health in Rats
Source: Foods. 2025 Feb 14;14(4):651. doi: 10.3390/foods14040651 (PMC11854857; doi:10.3390/foods14040651)
Supplement: Supplementary file 1 [file foods-14-00651-s001.zip › foods-3399687-supplementary.pdf]

Table S1 Test substance ankaflavin certificate of analysis.

**CERTIFICATE OF ANALYSIS**			
Product Name	Ankaflavin		
IUPAC Name	(3S,3aR,9aR)-9a-Methyl-3-octanoyl-6-[(1E)-1-propen-1-yl]-3a,4,8,9a-tetrahydro-2H-furo[3,2-g]isochromene-2,9(3H)-dione		
Synonyms	Not available		
CAS #	50980-32-0		
Molecular Formula	$C_{23}H_{30}O_5$	Molecular Weight	386.48
Data of analysis	18/12/2023	Retest Date	18/12/2028
Longterm Storage Conditions	Store at refrigerator (-20°C) for long term storage		
Shipping Conditions	Product is stable to be shipped at 0°C		

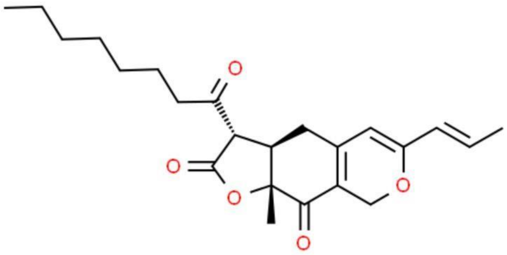

Test	Specification	Result
Appearance	Yellow powder	Yellow powder
Solubility	Soluble in Methanol	Soluble in Methanol
Purity by HPLC	Not less than 95%	97%
This Material complies.		

Table S2 Test substance monascin certificate of analysis.

CERTIFICATE OF ANALYSIS

Product Name	Monascin		
IUPAC Name	(3S,3aR,9aR)-3a,4,8,9a-Tetrahydro-9a-methyl-3-(1-oxohexyl)-6-(1E)-1- propenyl-2H-furo[3,2-g][2]benzopyran-2,9(3<I>H)-dione (3S,3aR,9aR)-3-Hexanoyl-9a-methyl-6-((E)-propenyl)-3a,4,8 ,9atetrahydro-3H-furo[3,2-g]isochromene-2,9-dione		
Synonyms	Not available		
CAS #	21516-68-7		
Molecular Formula	$C_{21}H_{26}O_5$	Molecular Weight	358.43
Data of analysis	18/12/2023	Retest Date	18/12/2028
Longterm Storage Conditions	Store at refrigerator (-20°C) for long term storage		
Shipping Conditions	Product is stable to be shipped at 0°C		

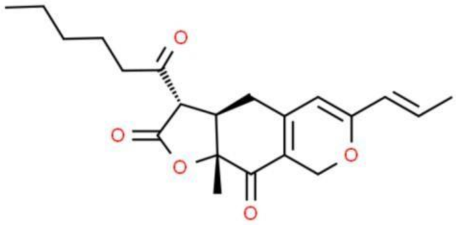

Test	Specification	Result
Appearance	Yellow powder	Yellow powder
Solubility	Soluble in Methanol	Soluble in Methanol
Purity by HPLC	Not less than 95%	99%
This Material complies.		

Table S3 Test substance monascinol certificate of analysis.

**CERTIFICATE OF ANALYSIS**			
Product Name	Monascinol		
IUPAC Name	(3S,3aR,9aR)-3-[(1R)-1-Hydroxyhexyl]-9a-methyl-6-[(1E)-1-propen-1-yl]-3a,4,8,9a-tetrahydro-2H-furo[3,2-g]isochromene-2,9(3H)-dione		
Synonyms	Not available		
CAS #	211244-19-1		
Molecular Formula	$C_{21}H_{28}O_5$	Molecular Weight	360.44
Data of analysis	18/12/2023	Retest Date	18/12/2028
Longterm Storage Conditions	Store at refrigerator (-20°C) for long term storage		
Shipping Conditions	Product is stable to be shipped at 0°C		
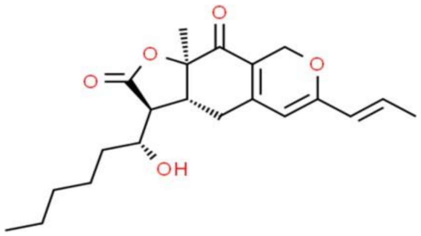			
Test	Specification	Result	
Appearance	Yellow powder	Yellow powder	
Solubility	Soluble in Methanol	Soluble in Methanol	
Purity by HPLC	Not less than 95%	98%	
This Material complies.			

Sample Information

Acquired by	: System Administrator
Sample Name	: 202230221-MS200-QC
Sample ID	:
Tray#	1
Vial#	11
Injection Volume	20
Data File	: 202230221-MS200-QC.lcd
Method File	: 2012.12.5 - OBB-33min-RD test.lcm
Batch File	: 20230221-RD-1.lcb
Report Format File	: DEFAULT.lsr
Date Acquired	: 2023/2/21 下午 10:41:35
Date Processed	: 2023/3/1 下午 04:47:37

Contour

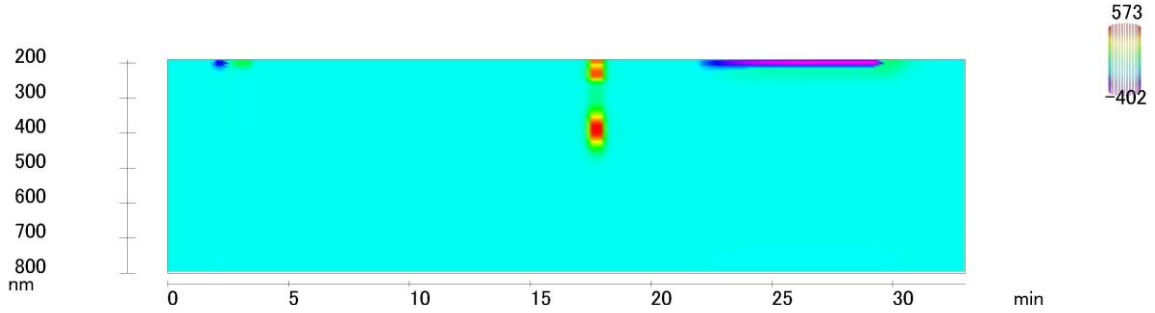

Chromatogram

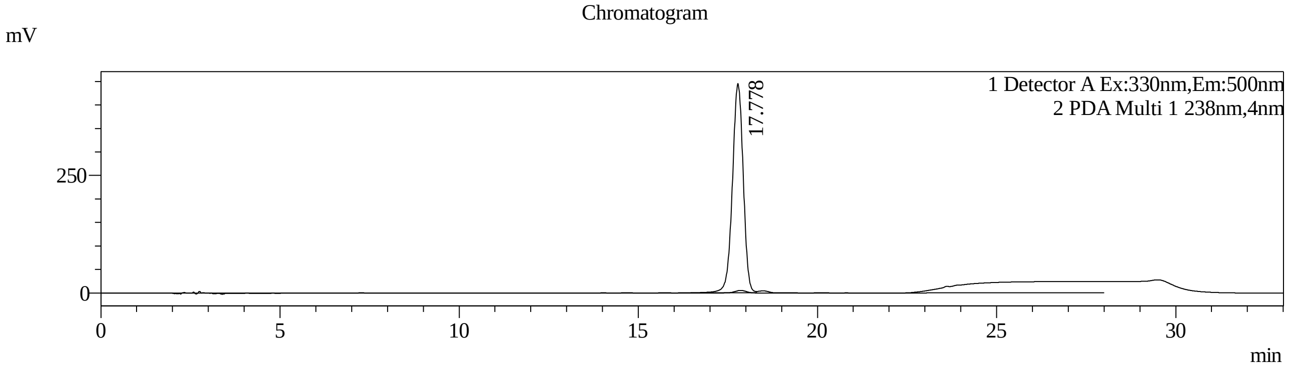

Peak Table

Detector A Ex:330nm,Em:500nm

Peak#	Ret. Time	Area	Height	Conc.	Unit	Mark	Name
Total							

PDA Ch1 238nm

Peak#	Ret. Time	Area	Height	Conc.	Unit	Mark	Name
1	17.778	9370292	445219	388.001	mg/L	M	MS
Total		9370292	445219				

Figure S1 HPLC analysis of monascin.

Sample Information

Acquired by	: System Administrator
Sample Name	: 202230221-AK200-RD
Sample ID	:
Tray#	1
Vial#	15
Injection Volume	20
Data File	: 202230221-AK200-RD.lcd
Method File	: 2012.12.5 - OBB-33min-RD test.lcm
Batch File	: 20230221-RD-1.lcb
Report Format File	: DEFAULT.lsr
Date Acquired	: 2023/2/22 上午 01:29:32
Date Processed	: 2023/3/1 上午 11:07:52

Contour

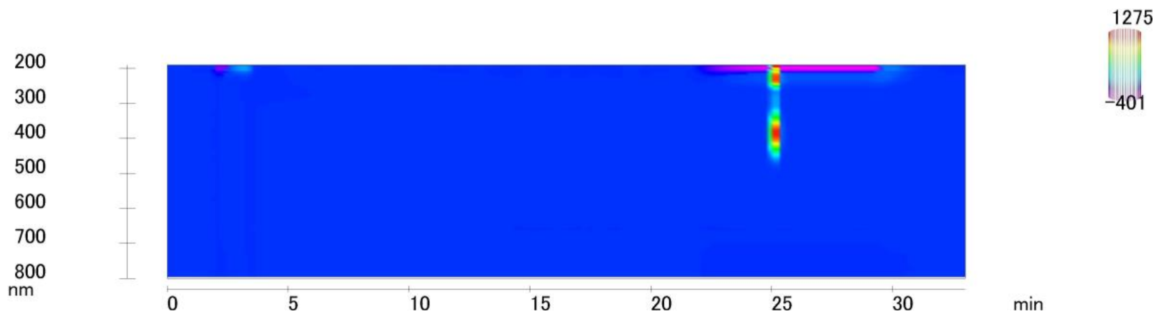

Chromatogram

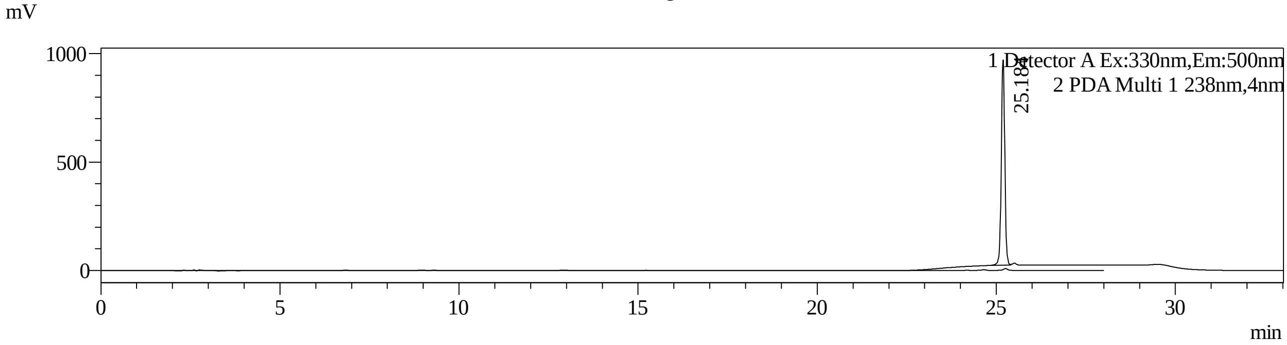

Peak Table

Detector A Ex:330nm,Em:500nm

Peak#	Ret. Time	Area	Height	Conc.	Unit	Mark	Name
Total							

PDA Ch1 238nm

Peak#	Ret. Time	Area	Height	Conc.	Unit	Mark	Name
1	25.184	6480849	945622	0.000		M	
Total		6480849	945622				

Figure S2 HPLC analysis of ankaflavin.

Sample Information

Acquired by : System Administrator
Sample Name : 20240103-MSol-200ppm
Sample ID :
Tray# 1
Vial# 3
Injection Volume 20
Data File : 20240103-MSol-200ppm.lcd
Method File : 2012.12.5 - OBB-33min-RD test.lcm
Batch File : 20240103-RD-1.lcb
Report Format File : DEFAULT.lsr
Date Acquired : 2024/1/4 上午 07:47:19
Date Processed : 2024/1/19 上午 10:42:17

Contour

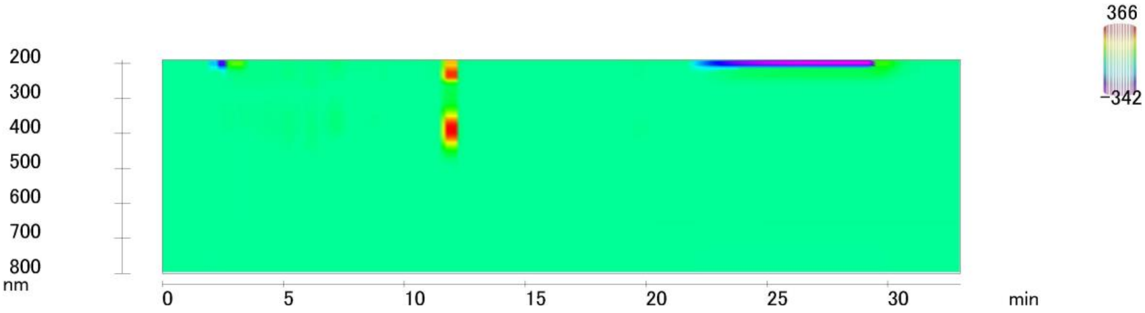

Chromatogram

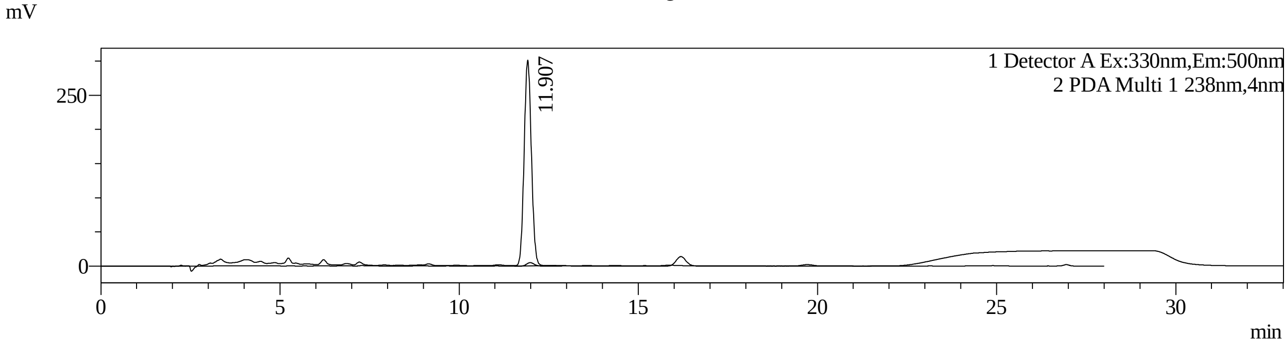

Peak Table

Detector A Ex:330nm,Em:500nm

Peak#	Ret. Time	Area	Height	Conc.	Unit	Mark	Name
Total							

PDA Ch1 238nm

Peak#	Ret. Time	Area	Height	Conc.	Unit	Mark	Name
1	11.907	4268555	301177	0.000			
Total		4268555	301177				

Figure S3 HPLC analysis of monascinol.
